# Integrative analysis of genomic sequencing data reveals higher prevalence of LRP1B mutations in lung adenocarcinoma patients with COPD

**DOI:** 10.1038/s41598-017-02405-9

**Published:** 2017-05-18

**Authors:** Dakai Xiao, Fuqiang Li, Hui Pan, Han Liang, Kui Wu, Jianxing He

**Affiliations:** 1 Department of Thoracic Surgery, The First Affiliated Hospital of Guangzhou Medical University, Guangzhou, 510120 China; 2 Research Center for Translational Medicine, The First Affiliated Hospital of Guangzhou Medical University, Guangzhou, 510120 China; 3 Guangzhou Institute of Respiratory Disease &State Key Laboratory for Respiratory Disease, Guangzhou, 510120 China; 4 Cancer Institute, BGI-Research, BGI-Shenzhen, Shenzhen 518083 China; 5 China National GeneBank-Shenzhen, BGI-Shenzhen, 518120 China; 6 Department of Biology, University of Copenhagen, DK-2200 Copenhagen N, Denmark

## Abstract

Both chronic Obstruction Pulmonary Disease (COPD) and lung cancer are leading causes of death globally. Although COPD and lung cancer coexist frequently, it is unknown whether lung cancer patients with COPD harbor distinct genomic characteristics compared to those without COPD. In this study, we retrospectively analyzed genomic sequencing data from 272 patients with lung adenocarcinoma (LUAD) and compared the genetic alterations in LUAD patients with and without COPD. Integrative analysis of whole-genome and exome sequencing data revealed that COPD and non-COPD groups showed high concordance in mutational burden and spectra. Notably, we also found that EGFR mutations were more prevalent in LUAD patients without COPD, whereas mutated LRP1B was more frequently observed in LUAD patients with COPD. In addition, multi-variable analysis with logistic regression demonstrated that mutation of LRP1B was a predictive marker for the presence of COPD in the patients with LUAD. Our analysis demonstrated for the first time the high concordance in genomic alterations between the tumors from LUAD patients with and without COPD. We also identified higher prevalence of LRP1B among the LUAD patients with COPD, which might help understand the underlying mechanisms which link COPD and lung cancer.

## Introduction

Chronic Obstruction Pulmonary Disease (COPD) and lung cancer are common lung diseases that coexist frequently. COPD is characterized by progressive airflow obstruction and chronic inflammation in the airways^1^. WHO estimates that COPD will become the third leading cause of death worldwide by 2030.

A number of epidemiological studies have demonstrated that the presence of COPD increased the risk of development of lung cancer^2–4^. Cigarette smoking is considered as a common cause of COPD and lung cancer, and the smokers with airflow obstruction are up to 6-fold more likely to develop lung cancer than those with normal lung function^5^. Despite the fact that cigarette smoking is a principal cause of both COPD and lung cancer, several population-based studies show that COPD confers the risk for lung cancer regardless of patients’ smoking history^2, 6–8^.

The high prevalence of lung cancer in COPD subjects suggests that there may be certain mechanisms linking COPD to lung cancer. In fact, several mechanisms including oxidative stress, genetic predisposition, epigenetic modifications and changes in inflammatory milieu and immune defenses, have been proposed to link the pathogenesis of COPD and development of lung cancer^2, 9, 10^. Genome-wide association studies (GWAS) have identified several single nucleotide polymorphisms (SNPs) which predispose to increased susceptibility to COPD and lung cancer such as SERPIN2, HHIP, FAM13A, IREB2, CHRNA3 and CHRNA5^2, 11^. Other studies also showed that inflammatory response factors, aberrant NF-κB activation and cytokine release, and high levels of CD8+ T cells mediated the link between COPD and lung cancer^12, 13^.

It is no doubt that understanding the mechanistic link between COPD and lung cancer would provide therapeutic and preventive benefit for the patients with COPD. However, the molecular mechanisms linking COPD with lung cancer development are far from clear, and the heterogeneous nature of lung cancer and COPD made it difficult to identify the mechanisms which linked COPD to lung cancer. It is also unknown whether LUAD patients with COPD harbor distinct genetic characteristics compared to those without COPD.

In this study, we retrospectively analyzed the genomic sequencing data from 272 LUAD patients, which was obtained from our previous study^14^. We correlated the clinical characteristics with genomic alterations, and found the high concordance of genomic alterations in the tumors from LUAD patients with or without COPD. Furthermore, we also found EGFR mutations were more prevalent in LUAD patients without COPD, whereas mutated LRP1B was more frequently observed in LUAD patients with COPD. To the best of our knowledge, our study revealed for the first time the high concordance of genomic alterations in the tumors from LUAD patients with COPD and non-COPD at genome-scale. These results also suggested that LUAD patients with COPD habored distinct somatic alterations in a few genes which can be exploited for personalized medical care for those patients.

## Results

### The clinicopathological characteristics of the LUAD patients with COPD

Among a total of 272 LUAD patients enrolled in this respective study, 60 subjects (22%) were diagnosed as COPD, and 212 subjects (78%) were classified into non-COPD group. As shown in Table 1, Pearson’s chi-square analysis revealed that LUAD patients with COPD were significantly associated with male sex, cigarette smoking, older age (64 years for COPD Vs 57 years for non-COPD, p < 0.001), and lower value of FEV1, FEV1% and FEV1/FVC (Table 1).Interestingly, LUAD patients with COPD also exhibited higher Lymphocyte/Monocyte Ratio (LMR) than non-COPD subjects. However, TNM stage, and counts of neutrophil cells, basophil cells and WBC in the peripheral blood did not differ significantly between two groups (Table 1).

**Table1:** Characteristics of 272 LUAD patients and their association with COPD status.

Characteristics	Overall	COPD	Non-COPD	*P*-value
	N = 272	n = 60	n = 212	
Age (y, mean ± SD)	58.64 ± 10.84	64.32	57.03	**<0.0001**
Gender				**0.0249**
Male	149	41	108	
Female	123	19	104	
Smoking				**0.0009**
No	157	23	134	
Yes	89	30	59	
Unknown	26	7	19	
WBC (×10^9^/L)	7.28 ± 2.39	7.63	7.19	0.667^†^
Neutrophil (×10^9^/L)	4.59 ± 2.05	4.9	4.5	0.262
Eosinophil (×10^9^/L)	0.21 ± 0.21	0.27	0.2	0.067
Basophil (×10^9^/L)	0.035 ± 0.019	0.036	0.034	0.678
Monocyte (×10^9^/L)	0.56 ± 0.32	0.61	0.5	0.130
Lymphocyte (×10^9^/L)	1.91 ± 0.65	1.83	1.93	0.361
Lymphocyte-Monocyte ratio	3.90 ± 1.70	3.51	4.01	**0.045**
CEA (ng/ml)	4.82 (0.33–1780)	39.48	10.8	0.337
CA125 (U/ml)	13.67 (2.04–526)	32.16	23.42	0.389
CA153 (U/ml)	13.15 (3.47–180.8)	15.88	18	0.252
NSE (ng/ml)	15.2 (8.5–63.0)	17.38	16.47	0.706
CYFRA21–1 (ng/ml)	2.7 (1.13–12.08)	3.25	3.02	0.594
FVC (%Pred)	97.68 ± 17.46	94.45 ± 20.97	98.60 ± 16.27	0.161
FEV1 (%Pred)	91.41 ± 20.30	71.64 ± 20.88	97.01 ± 16.28	**<0.0001**
FEV1/FVC	76.12 ± 10.82	60.17 ± 8.96	80.64 ± 5.91	**<0.0001**
FEV1 (value)	2.31 ± 0.65	1.82 ± 0.64	2.44 ± 058	**<0.0001**

^†^U-test.

### The genomic characteristics of LUAD patients with COPD

To explore the genomic alterations associated with COPD in LUAD, we firstly analyzed the whole-genome/exome sequencing data obtained by Wu *et al*. in 85 patients with spirometry data available^14^. Of these, 18 cases (21.2%) were diagnosed as COPD, and 67 cases (78.8%) were classified in non-COPD group. To determine if genomic differences existed between tumors from COPD and non-COPD group, we compared the mutational burden, spectrum and affected genes between these two groups. Negative binomial regression in univariate analysis revealed that the number of missense mutation was much higher in COPD patients compared to non-COPD patients (Table 2, COPD median = 88.5 Vs non-COPD median = 51, *p* = 0.021). Similarly, the number of nonsense mutation (Supplementary Table S1, COPD median = 8 vs non-COPD median = 4, p = 0.0358) and splice variant (COPD median = 2.5 vs non-COPD median = 2, p = 0.0371) was also significantly higher in COPD patients compared to non-COPD patients. Given the higher number of mutational burden among smokers and male patients in this study (Table 2) and previous studies^15, 16^, multivariable analysis with negative binomial regression was performed. After adjusting of age at diagnosis, gender and smoking status, no statistical difference in number of missense mutation was found between the COPD and non-COPD groups (Table 2), suggesting that mutational burden was not an independent factor associated with COPD status. We further performed the same analysis on the whole-exome sequencing data from the LUAD patients in The Cancer Genome Atlas (TCGA) cohort. Ninety-nine LUAD patients with spirometry data available were enrolled in this validation cohort. In contrasts to our cohort, most of these patients in TCGA cohort were from North America (detailed characteristics of these patients were summarized in Supplementary Table S2). Negative binomial regression in univariate analysis showed that no significant difference was observed in mutational burden between COPD and non-COPD group (Supplementary Table S3, COPD median = 195 vs non-COPD median = 203, p = 0.133) probably due to higher percentage of smokers in non-COPD group in TCGA cohort(64/75, 85.3%) compared to our cohort (23/62, 37.1%).

**Table2:** Univariate and multivariate analysis with negative binomial regression comparing the mutation counts by variables in patients with lung adenocarcinoma (n = 85).

Variable	No. of patients (n = 85)	No. of missense mutation median(range)	Univariate analysis p value	Multivariate analysis p value
Age, year			0.765	0.897
≤65	59	48 (3–787)		
>65	26		80.5 (19–236)	
Gender			**<0.0001**	**0.0002**
Male	52	92 (16–787)		
Female	33	34 (3–207)		
Smoking			**<0.0001**	**0.0310**
Yes	33	126 (16–787)		
No	47	46 (3–262)		
NA	5	—		
Stage				
I	18	39.5 (3–787)	(reference)	(reference)
II	16	74.5 (20–262)	0.879	0.3340
III	45	59 (16–690)	0.332	0.0940
IV	6	50.5 (37–144)	0.43	0.6670
NA		—		
COPD			**0.0211**	0.6560
Yes	18	88.5 (19–690)		
No	67	51.0 (3–787)		

NA, Not applicable.

**Figure 1:**
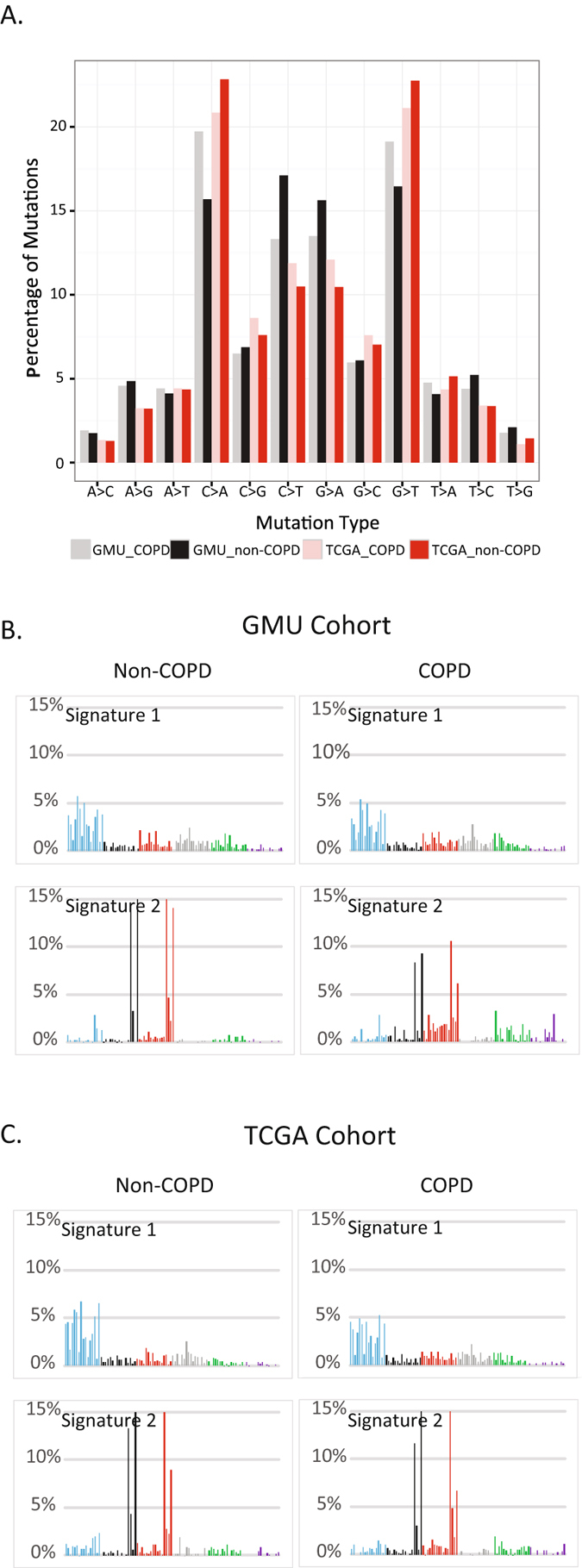
Mutational patterns of LUAD with and without COPD. (**A**) Comparison of mutational types and frequencies between LUAD with or without COPD in this study (Guangzhou Medical University (GMU) cohort) as well as in TCGA cohort. Mutational Signatures of LUAD with or with COPD derived from GMU cohort (**B**) and TCGA cohort (**C**). Signatures were displayed according to the 96-substitution classification, with x-axes showed mutation types and y-axes showed trinucleotide frequency of each mutation type.

We further illustrated the characteristics of mutation in patients from TCGA and our cohort according to their COPD status as shown in Fig. 1A. Similar mutational spectra were observed between COPD and non-COPD groups in both cohorts. Specifically, C:G → A:T transversions were the most frequent somatic substitutions, which was reported predominately in cigarette smoker, whereas C:G → T:A transitions were the most common point mutations which were found in never-smokers^16, 17^.

As COPD increased the risk for the development of lung cancer, we continued to explore whether COPD could leave distinct mutational patterns in the genome of LUAD. As mutational catalogues from exomes could also be used to decipher mutational signature^18^, we thus compared the mutation signatures between COPD and non-COPD patients using the whole-exome sequencing data as described previously^18^. Two highly confident mutational signatures were extracted from each group, showing no significant difference between COPD and non-COPD group. The first highly correlated signature (Fig. 1B,C, signature 1 in our cohort and TCGA cohort, Pearson Correlation >0.95) was predominated by C > A mutations and associated with cigarette smoking exposure. The second highly correlated signature (Pearson Correlation >0.91) was closely associated with over-activated member of APOBEC cytidinedeaminase^17^. As a validation, two similar mutation signatures were also generated from COPD and non-COPD group in TCGA cohort. Taken together, the mutational signature revealed that the tumors from LUAD patients with and without COPD exhibited similar mutational patterns.

### Somatic alterations in LUAD patients with COPD

**Figure 2:**
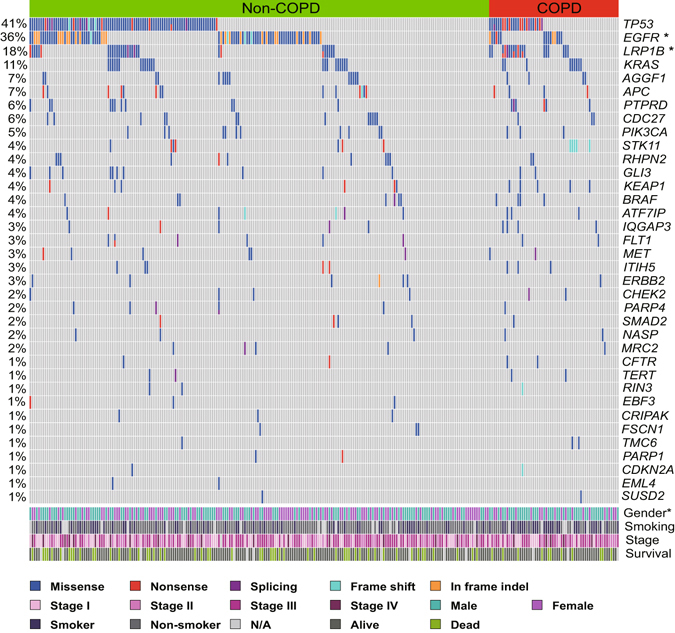
Recurrent Somatic mutations and their associations with clinical parameters in LUAD patients with or without COPD. Recurrently mutated genes and the mutant frequencies in the primary tumors with or without COPD from GMU cohort were shown. Gender, smoking status, tumor stages and survival were listed at the bottom according to the samples, as well as mutation types. Asterisks indicate genes predicted to be significantly mutated between COPD and non-COPD group (P < 0.05, Fisher’s exact test).

Several previous studies have demonstrated that mutation in EGFR was inversely associated with the presence of COPD in lung cancer^19, 20^. Although no significant difference was found in mutational signature between COPD and non-COPD group, we continued to focus on the genetic alterations in individual genes between two groups. To increase the statistical power, somatic alterations obtained from targeted sequencing data of another 187 cases was also included in this analysis. As shown in Fig. 2, Fisher’s exact test analysis revealed that among the frequently mutated genes, mutations in EGFR were more enriched in non-COPD patients (25.0% in COPD Vs 39.6% in non-COPD, p = 0.047), while mutations in LRP1B were more frequently observed in COPD (31.7% in COPD Vs 13.7% in non-COPD, p = 0.003). Consistent with previous study^21, 22^, COPD was not associated with KRAS mutation in our cohort (Fig. 2).

To exclude the influence of smoking, we continued to compare the genetic alterations between COPD and non-COPD patients among smokers and non-smokers, respectively. The subgroup analysis revealed that mutations in LRP1B were more frequently observed in COPD patients among non-smokers (21.7% in COPD Vs 8.2% in non-COPD, *p* = 0.06) and smokers (40% in COPD Vs 23.7% in non-COPD, *p* = 0.14) although the differences were not statistically significant largely due to the limited sample size in subgroup (Supplementary Fig. S1). This result suggested that mutation in LRP1B was closely correlated with COPD-associated lung cancer.

To validate these findings in an independent cohort, we analyzed the exomic sequencing data of tumors and normal control from the LUAD patients in TCGA cohort. However, as shown in Supplementary Fig. S2, there was no difference in mutational profile between COPD and non-COPD patients when we focused on the mutated genes.

### Multivariable analysis of risk factors associated with the presence of COPD in LUAD patients

In the univariate analysis, we found the presence of COPD in LUAD patients was associated with older age at diagnosis, cigarette smoking and male sex, LMR and higher prevalence of somatic mutation in LRP1B, lower prevalence of mutation in EGFR.

To identify the independent risk factors associated with the presence of COPD in LUAD, we performed multivariable analysis with logistic regression using sex, age at diagnosis, smoking status, LMR, EGFR and LRP1B mutation as covariates. The result showed LRP1B mutation remained as an independent risk factor associated with COPD in LUAD even after adjustment of age, gender and smoking status (Table 3, HR = 2.33, 95%CI: 1.04–5.21, *p* = 0.039). This result suggested that mutation in LRP1B might help distinguish between lung cancer in the presence and absence of COPD.

**Table 3:** Multivariate analysis of patient characteristics associated with COPD.

Variable	COPD		
	Odds Ratio	95%CI	*p*-value
Age	1.07	1.03–1.11	0.0004
Smoking, yes	2.56	1.16–5.85	0.022
LRP1B, mutated	2.33	1.04–5.21	0.039

### The association of COPD with overall survival of LUAD patients

To further investigate the association between COPD and overall survival in LUAD patients, we performed univariate analysis showing that the presence of COPD was not associated with overall survival of LUAD patients in our cohort (Supplementary Table S3). In contrast, gender, stage, preoperative LMR and levels of CA125 and CA153 were associated with worse overall survival in our cohort (Supplementary Table S4).

## Discussion

COPD and lung cancer are both common pulmonary diseases, and epidemiological studies show that COPD represents an independent risk factor for lung cancer. Although LUAD patients with COPD displayed distinct clinical characteristics such as older age, male sex and cigarette smoking, it is unknown whether lung cancer patients with COPD harbor distinct genomic characteristics compared to those without COPD. In this study, we aimed to identify the somatic genetic alterations that might distinguish between LUAD patients with and without COPD. Our analysis of genomic sequencing data showed the high concordance in mutational burden and spectrum between the LUAD patients with and without COPD. Moreover, we also identified EGFR and LRP1B were mutated at different frequencies in COPD and non-COPD groups.

COPD is characterized by more excessive inflammation and oxidative stress response when compared with lung cancer. It is unknown that whether there was difference in the mutational profiling of the tumors arose from different tumor microenvironments. Mutational signature analysis in this study suggested that there was no specific mutation pattern during the development of lung cancer associated with COPD. The high concordance in the mutational burden and spectra further suggested the inflammatory milieu surrounding the tumors cells did not generate new mutations in LUAD patients. This notion was supported by the recent study which reported that epithelial-mesenchymal transition (EMT) was an independent mediator driving changes in the tumor immune microenvironment in LUAD, but not a surrogate for mutational burden^23^. In contrast, previous integrative analysis of DNA methylation and transcriptome profiling demonstrated that the presence of COPD was associated with changes of methylation and expression in genes involved in immune response in non-small cell lung cancer (NSCLC)^24^.

Despite the high concordance in mutational pattern and burden between COPD and non-COPD groups, we still found different frequencies of mutations in EGFR and LRP1B genes among LUAD patients with and without COPD. In fact, lower mutation rate of EGFR in lung cancer associated-COPD has been found in several studies^19, 20^. However, the higher prevalence of LRP1B mutation in LUAD associated-COPD was reported for the first time by analyzing the genomic sequencing data. LRP1B was reported as a putative tumor suppressor gene, encoding a new member of low density lipoprotein receptor. The dysregulation of this gene was associated with cell migration, chemoresistance and worse clinical outcome in cancer^25, 26^. LRP1B was also identified as a potential driver gene and its mutation was significantly associated with cigarette smoking in LUAD^14^, while our results indicated that LRP1B mutation was associated with COPD independent of cigarette smoking. Given that COPD is an independent risk factor for lung cancer, this study suggested that early monitor of lung cancer should be performed in the patients with COPD. This finding also suggested that mutation in LRP1B could be a predictive biomarker that distinguished the LUAD patients in the presence and absence of COPD.

Meta-analysis demonstrated that the presence of COPD was associated with worse clinical outcome of lung cancer^27^. Even though we and other group demonstrated that lung cancer-associated with COPD preferentially harbored somatic mutations in a few genes, the tumors from COPD and non-COPD patients exhibited highly concordant genomic alterations, which might not explain the differences in clinical outcomes between these two groups.

We aimed to identify the genomic alterations in LUAD associated with COPD. Of note there were some limitations to this study. First, there were dramatic differences between our cohort and the validation cohort. The prevalence of smoker in COPD group from our cohort was relatively lower compared with the TCGA cohort (56.6% Vs 87.5%), but was comparable to that (59.4%) in another constitutive cohort of lung cancer patients with COPD from Korean population^19^. This might suggest the different etiological factors associated with COPD in different ethnic populations. In fact, the prevalence of COPD in China was significantly associated with poor ventilation in the kitchen and exposure to biomass fuels in addition to cigarette smoking, elder age^28^. Second, in TCGA cohort, most LUAD patients (423/522, 81.0%) were excluded for subsequent analysis due to missing spirometry data. The limited number of sample size made it difficult to identify the variants associated with COPD unbiasedly. Last, COPD is a quite heterogeneous obstructive lung disease, and the presence of emphysema confers increased risk of lung cancer. However, in this retrospective study, COPD patients were defined by airflow obstruction on the spirometry according to the Global Initiative of Chronic Obstructive Lung Disease (GOLD) guidelines, by which we could not discriminate between emphysema and chronic bronchitis predominant phenotypes. Therefore, further investigation in a large cohort is warranted to validate the distinct prevalence of mutations in EGFR and LRP1B among LUAD patients with COPD and clarify the impact of COPD on the survival of the patients with lung cancer.

All together, our integrative analysis of genomic sequencing data and clinicopathological information demonstrated that the LUAD patients with and without COPD harbored highly concordant genomic characteristics. Lung cancer-associated COPD might represent a distinct subtype of lung cancer associated with distinct molecular characteristics. Higher level of mutation in LRP1B and surrounded inflammatory microenvironment would be exploited as a therapeutic target and a diagnosis biomarker for LUAD patients with COPD.

## Materials and Methods

### Patient information

This study has been approved by Institutional Review Board of the First Affiliated Hospital of Guangzhou Medical University (Approval Number 2013–20) and conducted in accordance with the Declaration of Helsinki Principles. Written informed consent was obtained from all participants. The inclusion and exclusion criteria for the patients and specimens were described previously^14^. Briefly, three hundred and thirty-five LUAD patients whose tumors have been sequenced using whole-genome/exome or ultra targeted sequencing as previously were retrospectively analyzed. Of these, sixty-three patients who did not have the spirometry data available were excluded. Of the remaining 272 patients, whole genome or whole-exome sequencing was performed on the primary tumors and paired normal tissues from 85 cases, and targeted sequencing of 51 selected genes was performed in an additional 187 cases. The clinical data including gender, age at diagnosis, smoking status, TNM stage and spirometry data were extracted from electronic medical records. The COPD was diagnosed and classified according to the current Global Initiative of Chronic Obstructive Lung Disease (GOLD) guidelines. The LUAD patients with FEV1/FVC <70% (post-bronchodilator) were assigned to COPD group. Meanwhile, 99 out of 522 LUAD patients (19.0%) from TCGA with spirometry data available were also included in this study as an independent validation cohort.

### DNA sequencing data retrieval

The whole-genome/exome or targeted sequencing data of tumors and adjacent normal tissues from 272 LUAD patients and the clinical and demographic information were extracted from our previous study^14^ (All the sequencing data have been deposited at http://www.ebi.ac.uk/ega/).

DNA sequencing data of the tumors and normal controls and the corresponding clinical information from ninety-nine lung adenocarcinoma patients with spirometry data available in TCGA cohort were downloaded from gdac.broadinstitute.org.

### Mutation signature analysis of LUAD exomes

Mutational signature analysis was performed as described previously^14, 17, 18^. In brief, mutational catalogues from the genomic sequencing data of primary tumors derived from our cohort (n = 85) and TCGA cohort (n = 99) were used to decipher the mutational signatures. Pearson’s correlation analysis was performed to compare signature patterns between COPD and non-COPD groups. And the affected mutational process was determined by comparing the extracted signatures with signature set identified previously^17, 18^ (http://cancer.sanger.ac.uk/cosmic/signatures).

### Statistical analysis

Somatic variants from the LUAD patients in our cohort and TCGA cohort were extracted. Pearson’s Chi-square test or Fisher’s extract test were performed to compare the association between somatic alterations and clinical parameters such as gender, age, smoking and COPD status. Kaplan-Meier survival analysis was performed to estimate the overall survival with log rank test. The optimal cut-off value for Peripheral blood lymphocyte/monocyte ratio (LMR) was determined by Receiver-Operating Curve (ROC) analysis as described previously Multivariable analyses with logistic regression were carried out to determine the risk factor for the survival of LUAD with COPD. The p value < 0.05 is considered as statistically significant, and all the statistical tests were two-sided. The analyses were performed using SPSS16.0, R package and GraphPad Prism 6.0.

## Electronic supplementary material

Supplementary Information
