# Supplementary material for: Integrative analysis of genomic sequencing data reveals higher prevalence of LRP1B mutations in lung adenocarcinoma patients with COPD
Source: Sci Rep. 2017 May 18;7:2121. doi: 10.1038/s41598-017-02405-9 (PMC5437030; doi:10.1038/s41598-017-02405-9)
Supplement: Supplementary file 1 — Supplementary Information [file 41598_2017_2405_MOESM1_ESM.pdf]

Integrative Analysis of genomic sequencing data reveals higher prevalence of LRP1B mutations in lung adenocarcinoma patients with COPD

Dakai Xiao[1,2,3#], Fuqiang Li[4,5#], Hui Pan[1,2], Han Liang[4,5], Kui Wu*[4,5,6], Jianxing He*[1,2,3]

1. Department of Thoracic Surgery, The First Affiliated Hospital of Guangzhou Medical University, Guangzhou 510120, China
2. Research Center for Translational Medicine, The First Affiliated Hospital of Guangzhou Medical University, Guangzhou 510120, China
3. Guangzhou Institute of Respiratory Disease &State Key Laboratory for Respiratory Disease, Guangzhou 510120, China
4. Cancer Institute, BGI-Research, BGI-Shenzhen, Shenzhen 518083, China
5. China National GeneBank-Shenzhen, BGI-Shenzhen, 518120, China
6. Department of Biology, University of Copenhagen, DK-2200, Copenhagen N, Denmark

[#]These authors contributed equally to this work

Corresponding authors:

Jianxing He, M.D, Ph.D

Department of Thoracic Surgery

The First Affiliated Hospital of Guangzhou Medical University

E-mail: Drjianxing.he@gmail.com

Kui Wu, Ph.D

BGI-Shenzhen

E-mail: wukui@genomics.cn

Supplementary Figure Legend

Fig. S1:

Somatic mutations and their association with COPD in LUAD patients stratified by smoking status in GMU cohort. Mutated genes and mutant frequencies in the primary tumor with and without COPD from smokers (left panel) and non-smokers (right panel) were shown. Genders, smoking status, tumor stage, survival and mutation types were also listed at the bottom according to the samples

Fig.S2

Recurrent Somatic mutations and their association with COPD in LUAD patients. Mutated genes and the mutant frequencies in the primary tumors with or without COPD from TCGA cohort were shown. Genders, smoking status, tumor stage, survival and mutation types were listed at the bottom according to the samples.

Fig.S1

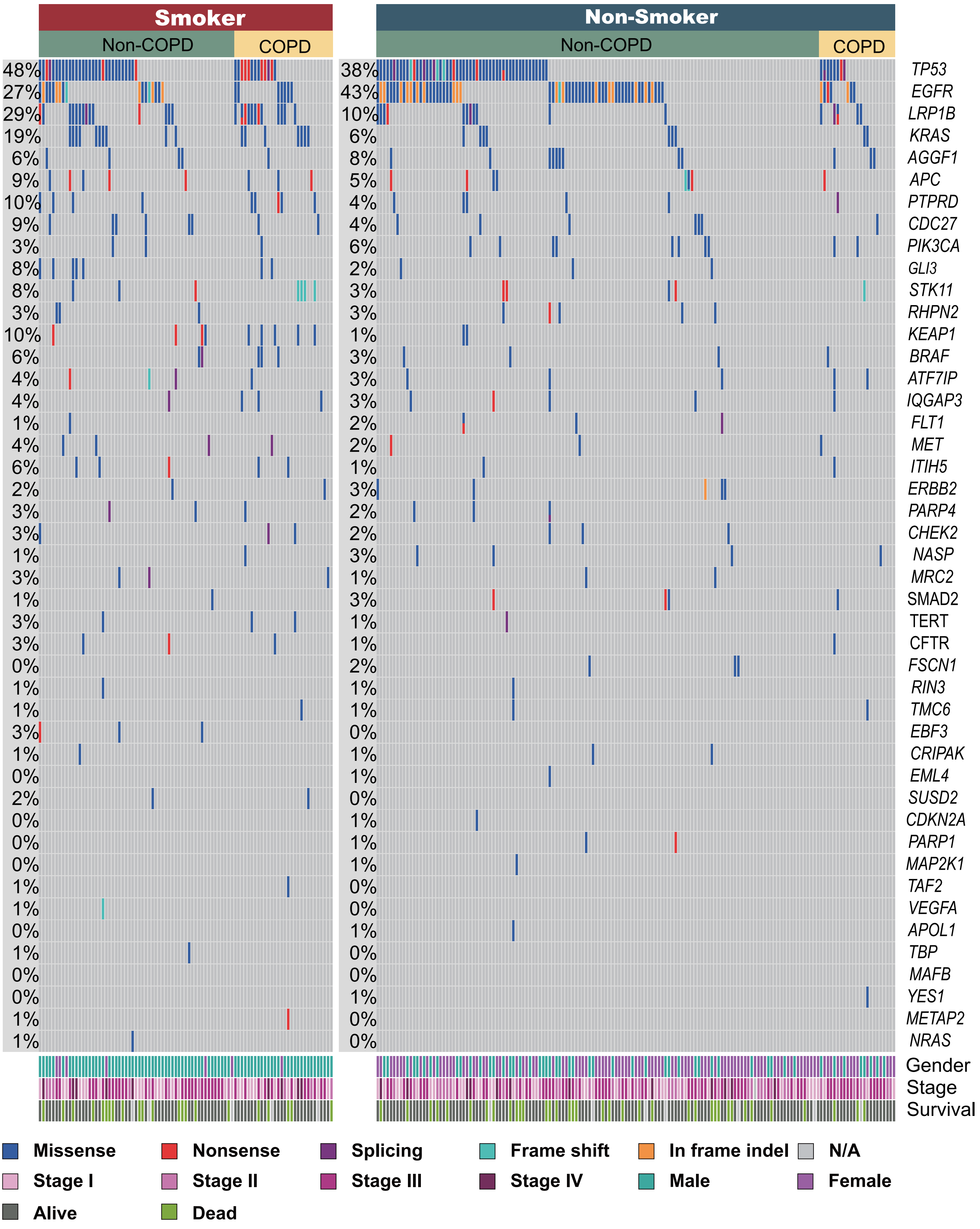

Smoker
Non-Smoker
Non-COPD
COPD
TP53
EGFR
LRP1B
KRAS
AGGF1
APC
PTPRD
CDC27
PIK3CA
GLI3
STK11
RHPN2
KEAP1
BRAF
ATF7IP
IQGAP3
FLT1
MET
ITIH5
ERBB2
PARP4
CHEK2
NASP
MRC2
SMAD2
TERT
CFTR
FSCN1
RIN3
TMC6
EBF3
CRIPAK
EML4
SUSD2
CDKN2A
PARP1
MAP2K1
TAF2
VEGFA
APOL1
TBP
MAFB
YES1
METAP2
NRAS
Gender
Stage
Survival
Missense
Nonsense
Splicing
Frame shift
In frame indel
N/A
Stage I
Stage II
Stage III
Stage IV
Male
Female
Alive
Dead

Fig.S2

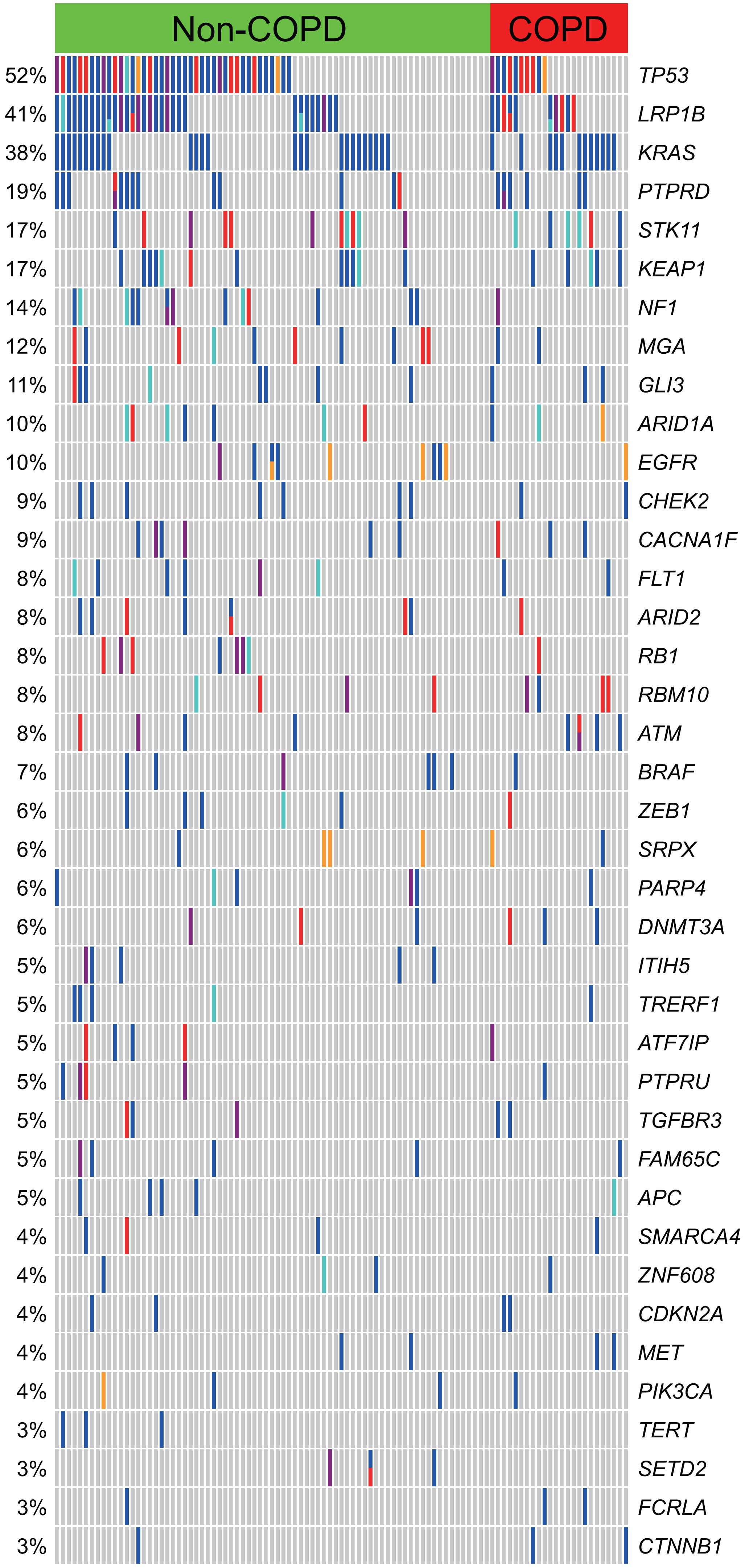
Non-COPD
COPD
52% TP53
41% LRP1B
38% KRAS
19% PTPRD
17% STK11
17% KEAP1
14% NF1
12% MGA
11% GLI3
10% ARID1A
10% EGFR
9% CHEK2
9% CACNA1F
8% FLT1
8% ARID2
8% RB1
8% RBM10
8% ATM
7% BRAF
6% ZEB1
6% SRPX
6% PARP4
6% DNMT3A
5% ITIH5
5% TRERF1
5% ATF7IP
5% PTPRU
5% TGFBR3
5% FAM65C
5% APC
4% SMARCA4
4% ZNF608
4% CDKN2A
4% MET
4% PIK3CA
3% TERT
3% SETD2
3% FCRLA
3% CTNNB1

Fig. S2
(Continued)

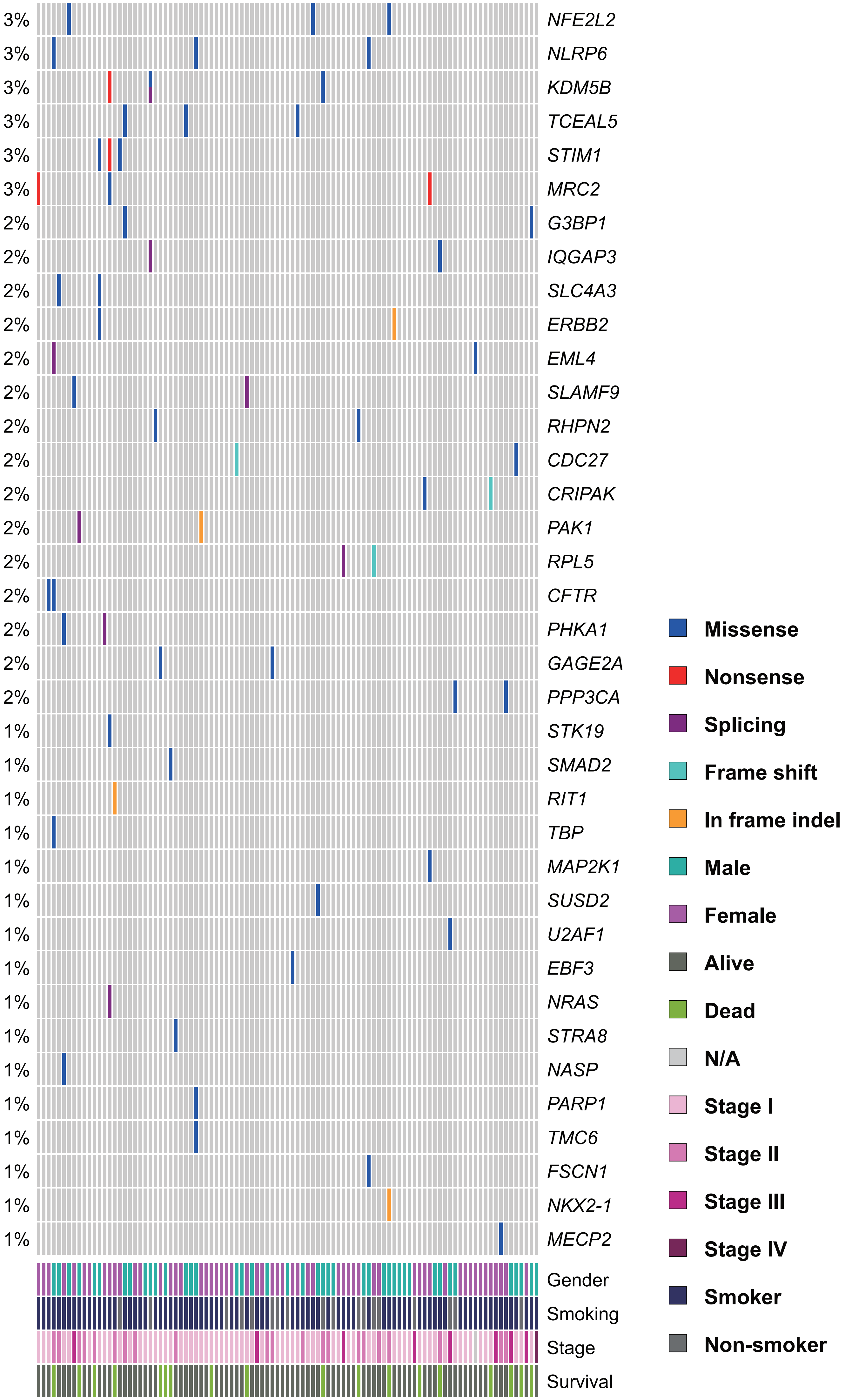

Supplementary Tables

Table S1

Univariate analysis with negative binomial regression comparing the counts of nonsense mutation and mutation in splicing site between COPD and non-COPD groups

	No.of patients	No.of Nonsense mutation median(range)	*p* value	No. of mutation in splicing site median(range)	*p* value
COPD			**0.0358**		**0.0371**
Yes	18	8(0-62)		2.5(0-28)	
No	67	4(0-65)		2(0-31)	

Table S2

Demographic and clinical information of LUAD patients from TCGA cohort

Variable	Overall (n=99)	COPD (n=24)	Non-COPD (n=75)	*P*-value
Age,mean±SD	66.66±9.49	68.83	65.96	0.134
Gender				0.75
Male	42	9	33	
Female	57	15	42	
Smoking				1
No	14	3	11	
Yes	85	21	64	
FEV1/FVC	80.24±21.62	53.13	88.92	<0.0001
Stage				0.057
Ⅰ	68	15	53	
Ⅱ	21	3	18	
Ⅲ	8	4	4	
Ⅳ	1	1	0	
NA	1			

Table S3

Univariate analysis with negative binomial regression comparing the counts of missense mutation between COPD and non-COPD groups in TCGA cohort

**Variable**	**No. of patients (n=99)**	**No.missense mutations median(range)**	Univariate analysis	Multivariate analysis
			p value	p value
Age，year			0.000649	0.0012
≤65	42	242.5(2-3738)		
>65	57	135(10-1073)		
Gender			0.254	0.4516
Male	42	195(10-3738)		
Female	57	203 (2-1819)		
Smoking			0.0169	0.0121
Yes	85	215(10-3738)		
No	9	191(45-240)		
NA	5	-		
Stage				
Ⅰ	68	206(10-3738)	(reference）	(reference）
Ⅱ	21	247(15-1819)	0.328	0.6198
Ⅲ	8	124.5(2-334)	0.036	0.4821
Ⅳ	1	51	0.074	0.0922
NA	1	-		
COPD			0.133	0.7166
Yes	24	195(51-903)		
No	75	203(2-3738)		

Table S4

Univariable analysis of factors associated with overall survival of LUAD patients

Variable	HR,95%CI	*P*-value
Age(year),≤65 vs >65	0.70(0.43-1.15)	0.158
Gender,male vs female	1.80(1.17-2.78)	**0.0078**
Smoking,yes vs no	0.97(0.61-1.56)	0.911
COPD,yes vs no	1.02(0.59-1.78)	0.946
Stage(Ⅰ vs Ⅱ vs Ⅲ vs Ⅳ)	-	**0.0014**
WBC,elevated vs normal	1.25(0.78-2.00)	0.356
Neutrophil, elevated vs normal	4.57(0.75-27.78)	0.099
Eosnophil, elevated vs normal	1.55(0.89-2.69)	0.122
Basophil, elevated vs normal	0.36(0.03-4.64)	0.434
Monocyte, elevated vs normal	0.60(0.22-1.69)	0.335
Lymphocyte, elevated vs normal	0.76(0.14-4.16)	0.75
LMR, High vs Low	0.57(0.36-0.91)	**0.018**
CEA, elevated vs normal	1.01(0.65-1.56)	0.975
CA125, elevated vs normal	2.16(1.07-4.34)	**0.031**
CA153, elevated vs normal	3.45(1.72-6.90)	0.00047
